# Prevalence of metabolic syndrome in scholars from Bucaramanga, Colombia: a population-based study

**DOI:** 10.1186/1471-2431-9-28

**Published:** 2009-04-21

**Authors:** Cristina Villa-Roel, Adriana Buitrago, Diana C Rodríguez, Diana J Cano, María P Martínez, Paul A Camacho, Álvaro J Ruiz, Álvaro E Durán

**Affiliations:** 1 Pediatric Research Area, Fundación Cardiovascular de Colombia, Floridablanca, Calle 155 A # 23 – 58, Urbanización el Bosque/Floridablanca/Santander, Colombia; 2 Department of Clinical Epidemiology and Biostatistics, Pontificia Universidad Javeriana, Bogotá, Carrera 7 No. 40 – 62 Bogotá DC, Colombia; 3 Department of Emergency Medicine, University of Alberta, 1G1.52 Walter C. Mackenzie Health Sciences Center, 8440 – 112 Street, Edmonton, Alberta, T6G 2B7, Canada

## Abstract

**Background:**

Obesity and metabolic syndrome are strongly associated with type 2 diabetes mellitus and cardiovascular diseases, thus the increasing trend in their prevalence among children and adolescents from developing countries requires a further understanding of their epidemiology and determinants.

**Methods and design:**

A cross-sectional study was designed to determine the prevalence of metabolic syndrome among 6–10 year-old children from Bucaramanga, Colombia. A two-stage random-cluster (neighborhoods, houses) sampling process was performed based on local city maps and local statistics. The study involves a domiciliary survey; including a comprehensive socio-demographic, nutritional and physical activity characterization of the children that participated in the study, followed by a complete clinical examination; including blood pressure, anthropometry, lipid profile determination, fasting glucose and insulin levels. The prevalence of metabolic syndrome will be determined using definitions and specific percentile cut-off points for this population. Finally, the association between components of metabolic syndrome and higher degrees of insulin resistance will be analyzed through a multivariable logistic regression model. This study protocol was designed in compliance with the Helsinki declaration and approved by the local ethics board. Consent was obtained from the children and their parents/guardians.

**Discussion:**

A complete description of the environmental and non-environmental factors underlying the burden of metabolic syndrome in children from a developing country like Colombia will provide policy makers, health care providers and educators from similar settings with an opportunity to guide primary and secondary preventive initiatives at both individual and community levels. Moreover, this description may give an insight into the pathophysiological mechanisms mediating the development of cardio-metabolic diseases early in life.

## Background

Cardiovascular diseases (CVD) are one of the major problems in public health and are responsible for more than 50% of all mortality in the western world [1]. CVD are not exclusive to developed countries. Nowadays, men, women and children from developing countries are recognized as populations at risk of developing CVD due to the nutritional, demographic, epidemiological and socioeconomic transitions they have been facing during the past few decades.

Persistent malnutrition, sedentary lifestyle and obesity have been associated with alterations in the processes that regulate the control of blood pressure and the action of insulin on the metabolism of carbohydrates and lipids. There is an urgent call to strategize health policies and intervention programs to tackle the factors influencing this phenomenon in the early stages of life [2]. Together these factors are known as the metabolic syndrome (MS) [3], a condition that is strongly associated with the development of type 2 diabetes mellitus (DM) and CVD [4-7].

### Metabolic Syndrome in children

Similar to adults, the prevalence of overweight and obese children and adolescents has shown an increasing trend [8-11]. Moreover, the association between obesity and the clustering of CVD risk factors early in life, and their persistence during adulthood has been explored by several groups [12-19]. Data pertaining to MS in children are scarce. This is in part due to the lack of a consensus on the definition of MS in this population and to the inclusion of children together with adolescents in most studies [20]. Furthermore, most of the epidemiological characteristics of MS in children have been studied in developed countries and little is known about this condition and its association with insulin resistance (IR) in children from developing countries. Several definitions for MS in children have been postulated [21-24]; however, the cut-off points for each of its components, as well as their diagnostic and prognostic validity, remain unclear [20]. More recent definitions of MS in children have tended to be age and ethnic-specific and to take into account developmental changes during the growing process [25,26]. It is well recognized that IR constitutes a crucial element linking the presence of cardiovascular risk factors and the development of CVD [27]. The Homeostasis Model Assessment (HOMA) index is one of the surrogate methods used for the evaluation of insulin sensitivity, and has proven to be strongly associated with endothelial dysfunction and the future development of DM and CVD [28].

Both, genetic and environmental factors interact and contribute to the burden of MS; however, environmental factors seem to play a predominant role. The most commonly described factors involved in the development of MS include: rapid nutritional transition, rural-to-urban migration, adoption of a sedentary lifestyle and maternal-fetal related conditions [29]. There is now a general agreement that all these elements need to be carefully analyzed from local, culturally-sensitive and cost-effective perspectives with the final goal of establishing globally acceptable criteria for the early detection and intervention of MS. Moreover, strategies for the management of MS require the integration of multiple stakeholders such as government and nongovernmental policy makers, interdisciplinary health care providers and educational organizations.

The aim of this study is to determine the prevalence of MS in 6–10 year-old children from Bucaramanga, Colombia, based on sex and age adjusted percentile cut-off points for the different anthropometric, hemodynamic and biochemical parameters in this population. The secondary objectives of this study involve a description of the children's socio-demographic, nutritional and physical activity profile and the analysis of the association between the proposed components of MS and higher degrees of IR (using the HOMA index).

## Methods and design

### Design

Cross-sectional and population-based study.

### Eligibility

Children, 6–10 years old, who had lived in Bucaramanga, Colombia during the last 12 months were eligible. Those with a history of menarche and/or Tanner stage ≥ 2, prior diagnosis of DM or other endocrine disorder; and/or previous treatment with hormones or steroids (except for sodium levothyroxine) during the last month were excluded.

### Sample size and method of recruitment

**Figure 1:**
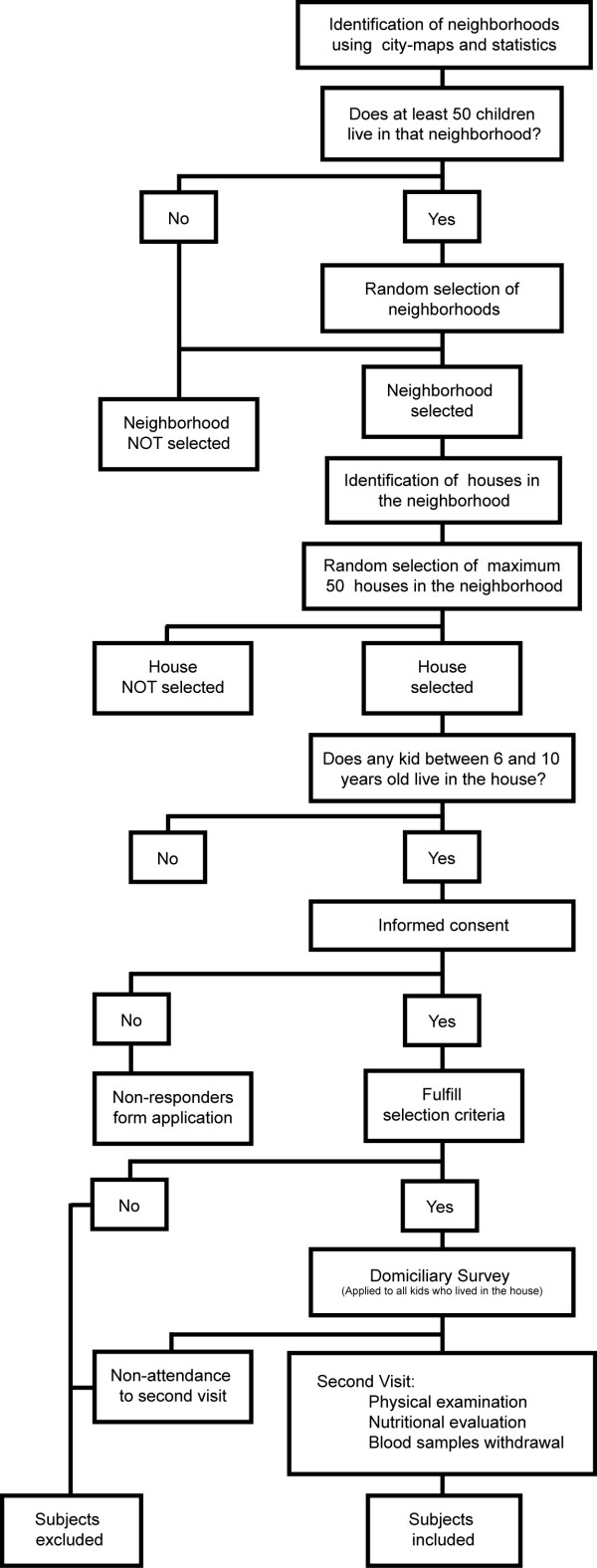
**Study flow**.

A sample size of 1282 children was calculated from the total 6–10 year-old population from Bucaramanga (N = 40,793), Colombia. We based this calculation on an expected prevalence of MS in children of 8%, an absolute precision between 1–5%, a confidence level of 95% and a design effect of 2. Using local city maps and local statistics [30,31], a two-stage sampling process was performed (Figure 1). In the first stage, a random selection of neighborhoods that had at least 50 children (clusters) was made. In the second stage, a random selection of a maximum of 50 houses in each neighborhood was completed. All children who fulfilled the inclusion criteria in each of the selected houses were invited to participate in the study.

### Data collection

During 16 months (July 2006–October 2007), a domiciliary survey for socio-demographic, nutritional and physical activity data collection was administered to the children's caregivers by trained research assistants. On a second visit, a complete physical examination (including blood pressure and anthropometric measurements) and blood withdrawal (for the determination of lipid profile, fasting glucose and insulin levels), were performed by trained physicians and nutritionists at the "Fundación Cardiovascular de Colombia" (FCV).

### Study variables

Demographic factors such as age, sex, ethnicity (visually assessed by a trained research assistant), educational level, and socioeconomic status were collected. Health care availability was confirmed by checking children's health insurance cards. Children's and family history were obtained directly from their caregivers and whenever possible confirmed by legal documents (e.g. birth certificate and medical records).

Smoking status was categorized as active and passive exposure to cigarettes. Nutritional habits were collected through a 24 hour recall. Physical activity was assessed by asking the time (hours per day) the children spent doing several activities such as sleeping, studying (both, at school and home), doing moderate physical activity (e.g. walking, biking, skating, playing in a pool, gymnastics, ballet or dancing; playing at home or in a park; playing an instrument), doing vigorous physical activities (running, carrying heavy objects, biking and skating with considerable speed, practicing competitive sports or dancing, playing football, basketball or volleyball) and spending in front of screens (e.g. television, computers or videogames), during one day of the week and weekend. Physical or neurological deficits were recorded if they were referred by the caregivers.

Children's heart and respiratory rates were measured in a comfortable condition after a 10-min resting period. Blood pressure was measured three times (with 5 minutes between each) with their arm supported at the level of the heart and seated quietly, using a automated blood pressure monitor (Dinamap Pro 100; GE Medical Systems Information Technologies, Inc., Milwaukee, Wisconsin) and following the recommendations of the National High Blood Pressure Education Program (NHBPEP) Working Group on Children and Adolescents [32]. Percentiles of blood pressure according to the percentiles of weight/age were determined [33,34].

Anthropometric variables (weight, height, skin-folds, waist and hip circumferences) were measured in duplicate by two independent nutritionists, in the morning after urine elimination, with the child using light clothing and no shoes [34,35]. The Body Mass Index (BMI) was calculated according to the CDC recommendations [36,37]. Percentiles of weight/age and of BMI were also determined [38].

Under fasting conditions (at least 10 hours), blood samples were taken from the antecubital vein. Glycemia and Lipid Profile were immediately quantified by a routine colorimetric method (Biosystems BTS-303 Photometric, Spain). Part of the samples were stored at -80°C, and insulin determinations assessed by electrochemiluminescence immunoassay "ECLIA" technique (Roche Elecsys 1010/2010 and MODULAR ANALYTICS E170 (Elecsys module) immunoassay analyzers) in one assay at the end of the study. The HOMA Index was obtained from a mathematical model using the following formula: (22.5/[I_F_*G_F_]) - (I_F_: Fasting insulin levels in IU/L, G_F_: Fasting glucose levels in mmol/L) [39].

### Data quality and statistical analyses

Study forms were reviewed to ensure they were complete before data entry. Data were entered by duplicate into Microsoft Excel spreadsheets (Microsoft Corp., Redmond, Wash.) and compared using Epi-Info 2000. Any discrepancy was corrected using the original study forms. The phase of data analysis is now in progress. Bivariable analyses for dichotomous and continuous variables will be performed by chi-square test, t-tests or Mann-Whitney tests, respectively. The association between components of MS and the higher quintile of the HOMA index will be analyzed through a logistic regression model considering adjustments by clustering effect. All p-values will be two-tailed, with p < 0.01 considered statistically significant. Data will be analyzed using Stata Statistical Software^®^: Release 10.0. (College Station, TX: Stata Corporation). Nutritional data will be analyzed using the software *Ceres *version 1.02 "FAO 1998" (adapted version for Latin America and the Caribbean).

### Logistical considerations

During the recruitment phase we faced several security issues. In order to assure that the sampling strategies were not violated we asked for support from community leaders and the local police department. We organized several promotion and health campaigns in tolerance zones in order to explain the purposes of the study, introduce the study personnel to the community and to agree on areas in which the survey and clinical examinations could be performed.

### Ethical considerations

The study protocol was designed in compliance with the Helsinki declaration and approved by the Research Ethics Board from the FCV. Consent was obtained from all participants and from their parents/guardians. Additionally, all children gave their verbal assent.

## Discussion

Environmental and non-environmental factors underlying MS in developing countries could be different to those described in other populations. Population-based research on this topic needs to take in to account the peculiarities and limited resources in these settings in order to preserve methodological rigor. Despite facing some security and logistical problems, a rigorous sampling method, data collection and management were completed during the first phase of this study. To our knowledge, this project constitutes the first population-based study of cardiovascular risk factors in Colombian scholars.

We strongly believe that the results of this study will help establish globally acceptable criteria for the early detection and intervention of MS in children. Moreover, they will provide policy makers, health care providers and educators from a developing country like Colombia with an opportunity to guide primary and secondary preventive initiatives at individual and community levels.

## Abbreviations

**BMI**: denotes body mass index; **CVD**: cardiovascular diseases; **DM**: diabetes mellitus; **FCV**: Fundación Cardiovascular de Colombia; **HOMA**: homeostasis model assessment; **MS**: metabolic syndrome; **NHBPEP**: National High Blood Pressure Education Program

## Competing interests

The authors declare that they have no competing interests.

## Authors' contributions

CVR was responsible for the study design, coordinated the study conduct and will be responsible for the analyses and manuscript production. AB, DCR and DJC coordinated the study personnel, the recruitment phase and were responsible for data management. MPM and PAC contributed to the study design and to the submission for funding. Finally, AJR and AED participated in the study design and supported the group in the submission for funding.

## Pre-publication history

The pre-publication history for this paper can be accessed here:


